# The History of Nanoscience and Nanotechnology: From Chemical–Physical Applications to Nanomedicine

**DOI:** 10.3390/molecules25010112

**Published:** 2019-12-27

**Authors:** Samer Bayda, Muhammad Adeel, Tiziano Tuccinardi, Marco Cordani, Flavio Rizzolio

**Affiliations:** 1 Department of Chemistry, Faculty of Sciences, Jinan University, Tripoli 818, Lebanon; 2 Pathology Unit, Centro di Riferimento Oncologico di Aviano (CRO) IRCCS, 33081 Aviano, Italy; muhammad.adeel@unive.it; 3 PhD School in Science and Technology of Bio and Nanomaterials, University Ca’ Foscari of Venice, 30170 Venice, Italy; 4 Department of Pharmacy, University of Pisa, 56126 Pisa, Italy; tiziano.tuccinardi@farm.unipi.it; 5 Instituto Madrileño de Estudios Avanzados en Nanociencia (IMDEA Nanociencia), 28049 Madrid, Spain; marco.cordani@imdea.org; 6 Department of Molecular science and Nanosystems, University Ca’ Foscari of Venice, 30170 Venice, Italy

**Keywords:** nanoscience, nanotechnology, nanomaterials, nanoparticles, nanomedicine

## Abstract

Nanoscience breakthroughs in almost every field of science and nanotechnologies make life easier in this era. Nanoscience and nanotechnology represent an expanding research area, which involves structures, devices, and systems with novel properties and functions due to the arrangement of their atoms on the 1–100 nm scale. The field was subject to a growing public awareness and controversy in the early 2000s, and in turn, the beginnings of commercial applications of nanotechnology. Nanotechnologies contribute to almost every field of science, including physics, materials science, chemistry, biology, computer science, and engineering. Notably, in recent years nanotechnologies have been applied to human health with promising results, especially in the field of cancer treatment. To understand the nature of nanotechnology, it is helpful to review the timeline of discoveries that brought us to the current understanding of this science. This review illustrates the progress and main principles of nanoscience and nanotechnology and represents the pre-modern as well as modern timeline era of discoveries and milestones in these fields.

## 1. Definition of Nanoscience and Nanotechnology

**Figure 1:**
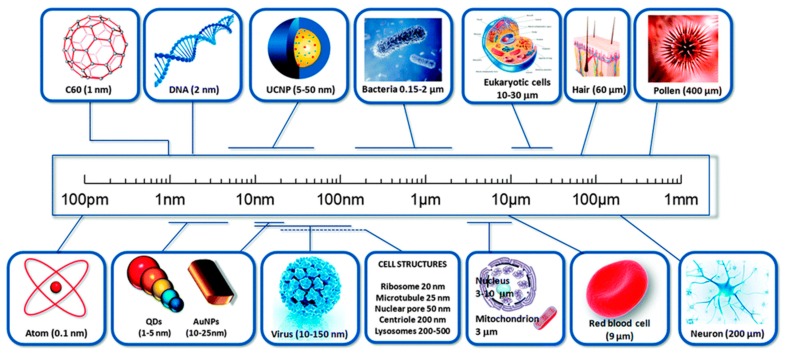
A comparison of sizes of nanomaterial. Reproduced with permission from reference [2].

The prefix ‘nano’ is referred to a Greek prefix meaning ‘dwarf’ or something very small and depicts one thousand millionth of a meter (10^−9^ m). We should distinguish between nanoscience, and nanotechnology. Nanoscience is the study of structures and molecules on the scales of nanometers ranging between 1 and 100 nm, and the technology that utilizes it in practical applications such as devices etc. is called nanotechnology [1]. As a comparison, one must realize that a single human hair is 60,000 nm thickness and the DNA double helix has a radius of 1 nm (Figure 1) [2]. The development of nanoscience can be traced to the time of the Greeks and Democritus in the 5th century B.C., when scientists considered the question of whether matter is continuous, and thus infinitely divisible into smaller pieces, or composed of small, indivisible and indestructible particles, which scientists now call atoms.

Nanotechnology is one of the most promising technologies of the 21st century. It is the ability to convert the nanoscience theory to useful applications by observing, measuring, manipulating, assembling, controlling and manufacturing matter at the nanometer scale. The National Nanotechnology Initiative (NNI) in the United States define Nanotechnology as “a science, engineering, and technology conducted at the nanoscale (1 to 100 nm), where unique phenomena enable novel applications in a wide range of fields, from chemistry, physics and biology, to medicine, engineering and electronics” [3]. This definition suggests the presence of two conditions for nanotechnology. The first is an issue of scale: nanotechnology is concerned to use structures by controlling their shape and size at nanometer scale. The second issue has to do with novelty: nanotechnology must deal with small things in a way that takes advantage of some properties because of the nanoscale [4].

We should distinguish between nanoscience and nanotechnology. Nanoscience is a convergence of physics, materials science and biology, which deal with manipulation of materials at atomic and molecular scales; while nanotechnology is the ability to observe measure, manipulate, assemble, control, and manufacture matter at the nanometer scale. There are some reports available, which provided the history of nanoscience and technology, but no report is available which summarize the nanoscience and technology from the beginning to that era with progressive events. Therefore, it is of the utmost requirements to summarize main events in nanoscience and technology to completely understand their development in this field.

## 2. The Imaginative Pioneers of Nanotechnology

The American physicist and Nobel Prize laureate Richard Feynman introduce the concept of nanotechnology in 1959. During the annual meeting of the American Physical Society, Feynman presented a lecture entitled “There’s Plenty of Room at the Bottom” at the California Institute of Technology (Caltech). In this lecture, Feynman made the hypothesis “Why can’t we write the entire 24 volumes of the Encyclopedia Britannica on the head of a pin?”, and described a vision of using machines to construct smaller machines and down to the molecular level [5]. This new idea demonstrated that Feynman’s hypotheses have been proven correct, and for these reasons, he is considered the father of modern nanotechnology. After fifteen years, Norio Taniguchi, a Japanese scientist was the first to use and define the term “nanotechnology” in 1974 as: “nanotechnology mainly consists of the processing of separation, consolidation, and deformation of materials by one atom or one molecule” [6]. 

After Feynman had discovered this new field of research catching the interest of many scientists, two approaches have been developed describing the different possibilities for the synthesis of nanostructures. These manufacturing approaches fall under two categories: top-down and bottom-up, which differ in degrees of quality, speed and cost.

The top-down approach is essentially the breaking down of bulk material to get nano-sized particles. This can be achieved by using advanced techniques such as precision engineering and lithography which have been developed and optimized by industry during recent decades. Precision engineering supports the majority of the micro-electronics industry during the entire production process, and the high performance can be achieved through the use of a combination of improvements. These include the use of advanced nanostructure based on diamond or cubic boron nitride and sensors for size control, combined with numerical control and advanced servo-drive technologies. Lithography involves the patterning of a surface through exposure to light, ions or electrons, and the deposition of material on to that surface to produce the desired material [7].

The bottom-up approach refers to the build-up of nanostructures from the bottom: atom-by-atom or molecule-by-molecule by physical and chemical methods which are in a nanoscale range (1 nm to 100 nm) using controlled manipulation of self-assembly of atoms and molecules. Chemical synthesis is a method of producing rough materials which can be used either directly in product in their bulk disordered form, or as the building blocks of more advanced ordered materials. Self-assembly is a bottom-up approach in which atoms or molecules organize themselves into ordered nanostructures by chemical-physical interactions between them. Positional assembly is the only technique in which single atoms, molecules or cluster can be positioned freely one-by-one [7].

**Figure 2:**
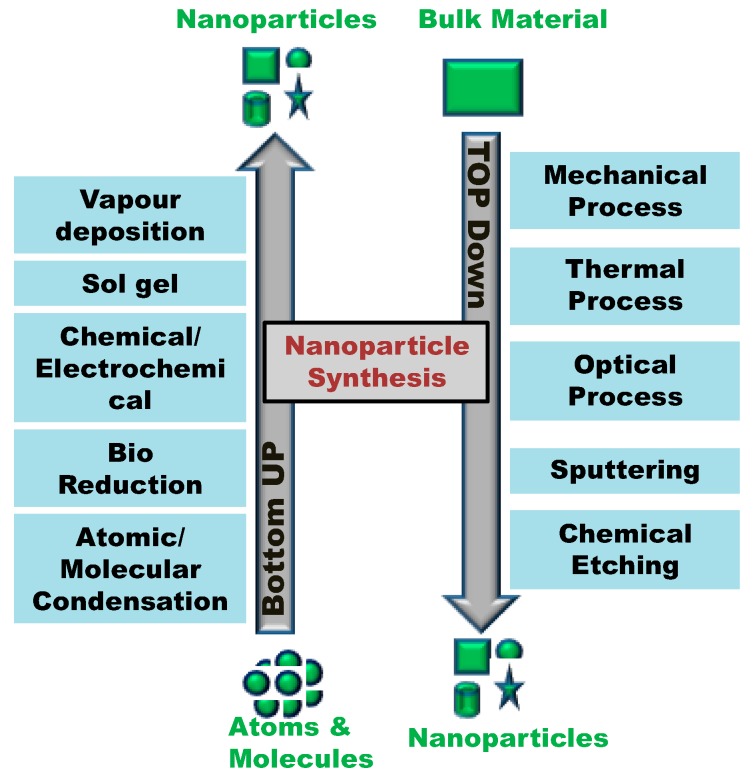
The concept of top down and bottom up technology: different methods for nanoparticle synthesis.

The general concept of top down and bottom up and different methods adopted to synthesized nanoparticles by using these techniques are summarized in Figure 2. In 1986, K. Eric Drexler published the first book on nanotechnology “Engines of Creation: The Coming Era of Nanotechnology”, which led to the theory of “molecular engineering” becoming more popular [8]. Drexler described the build-up of complex machines from individual atoms, which can independently manipulate molecules and atoms and thereby produces self-assembly nanotructures. Later on, in 1991, Drexler, Peterson and Pergamit published another book entitled “Unbounding the Future: the Nanotechnology Revolution” in which they use the terms “nanobots” or “assemblers” for nano processes in medicine applications and then the famous term “nanomedicine” was used for the first time after that [9].

## 3. History of Nanotechnology

**Figure 3:**
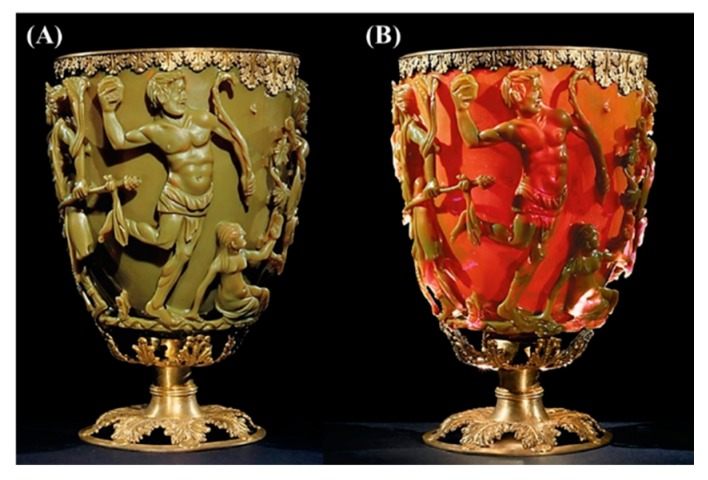
The Lycurgus cup. The glass appears green in reflected light (**A**) and red-purple in transmitted light (**B**). Reproduced with permission from reference [10].

Nanoparticles and structures have been used by humans in fourth century AD, by the Roman, which demonstrated one of the most interesting examples of nanotechnology in the ancient world. The Lycurgus cup, from the British Museum collection, represents one of the most outstanding achievements in ancient glass industry. It is the oldest famous example of dichroic glass. Dichroic glass describes two different types of glass, which change color in certain lighting conditions. This means that the Cup have two different colors: the glass appears green in direct light, and red-purple when light shines through the glass (Figure 3) [10].

**Figure 4:**
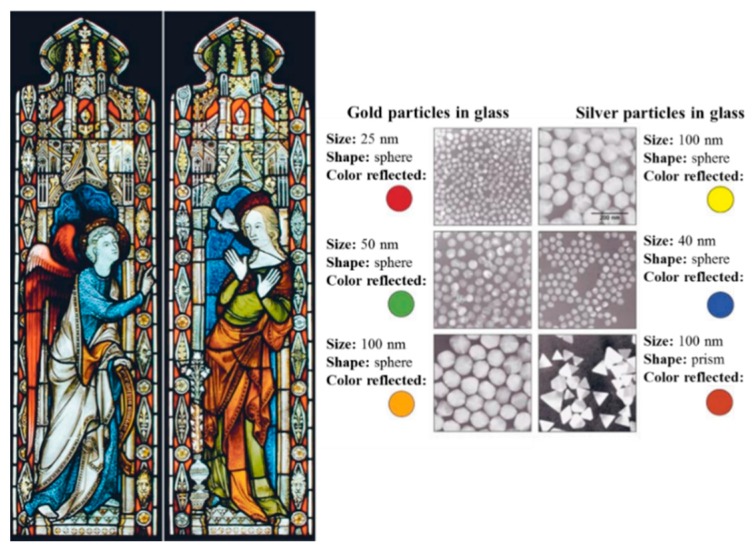
Effect of nanoparticles on the colors of the stained glass windows. Reproduced with permission from reference [14].

In 1990, the scientists analyzed the cup using a transmission electron microscopy (TEM) to explain the phenomenon of dichroism [11]. The observed dichroism (two colors) is due to the presence of nanoparticles with 50–100 nm in diameter. X-ray analysis showed that these nanoparticles are silver-gold (Ag-Au) alloy, with a ratio of Ag:Au of about 7:3, containing in addition about 10% copper (Cu) dispersed in a glass matrix [12,13]. The Au nanoparticles produce a red color as result of light absorption (~520 nm). The red-purple color is due to the absorption by the bigger particles while the green color is attributed to the light scattering by colloidal dispersions of Ag nanoparticles with a size > 40 nm. The Lycurgus cup is recognized as one of the oldest synthetic nanomaterials [1]. A similar effect is seen in late medieval church windows, shining a luminous red and yellow colors due to the fusion of Au and Ag nanoparticles into the glass. Figure 4 shows an example of the effect of these nanoparticles with different sizes to the stained glass windows [14].

During the 9th–17th centuries, glowing, glittering “luster” ceramic glazes used in the Islamic world, and later in Europe contained Ag or copper (Cu) or other nanoparticles [15]. The Italians also employed nanoparticles in creating Renaissance pottery during 16th century [16]. They were influenced by Ottoman techniques: during the 13th–18th centuries, to produce “Damascus” saber blades, cementite nanowires and carbon nanotubes were used to provide strength, resilience, and the ability to hold a keen edge [17]. These colors and material properties were produced intentionally for hundreds of years. Medieval artists and forgers, however, did not know the cause of these surprising effects.

**Figure 5:**
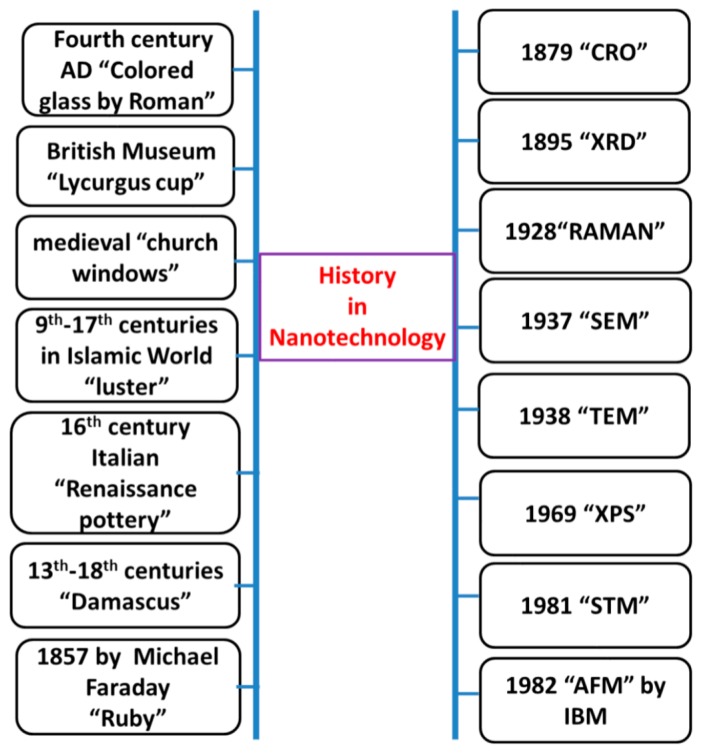
Progresses in Nanotechnology.

In 1857, Michael Faraday studied the preparation and properties of colloidal suspensions of “Ruby” gold. Their unique optical and electronic properties make them some of the most interesting nanoparticles. Faraday demonstrated how gold nanoparticles produce different-colored solutions under certain lighting conditions [18]. The progression in nanotechnology due to the blessings of nanoscience are summarized in the Figure 5.

## 4. Modern Era of Nanotechnology

**Figure 6:**
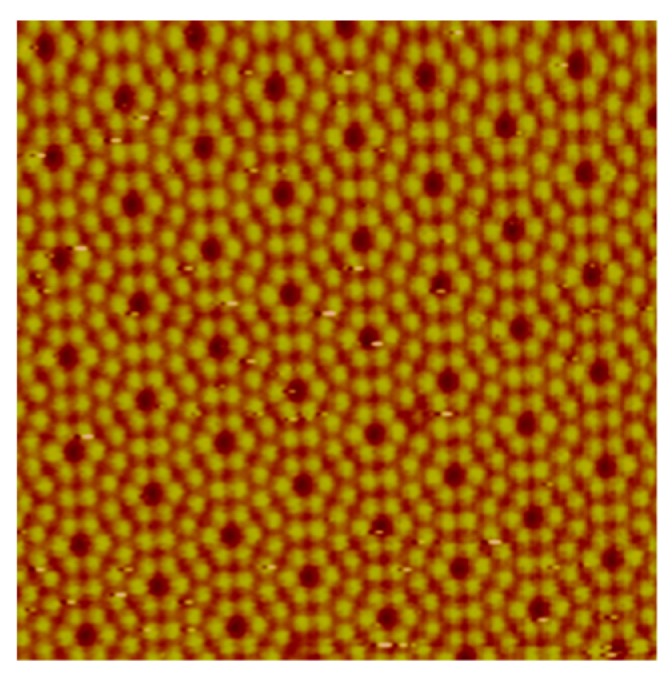
STM image of the Si(111)-7 × 7 reconstructed surface showing atomic scale resolution of the top-most layer of silicon atoms. Reproduced with permission from reference [22].

There was a progress in nanotechnology since the early ideas of Feynman until 1981 when the physicists Gerd Binnig and Heinrich Rohrer invented a new type of microscope at IBM Zurich Research Laboratory, the Scanning Tunneling Microscope (STM) [19,20]. The STM uses a sharp tip that moves so close to a conductive surface that the electron wave functions of the atoms in the tip overlap with the surface atom wave functions. When a voltage is applied, electrons “tunnel” through the vacuum gap from the atom of the tip into the surface (or vice versa). In 1983, the group published the first STM image of the Si(111)-7 × 7 reconstructed surface, which nowadays can be routinely imaged as shown in Figure 6 [21,22].

**Figure 7:**
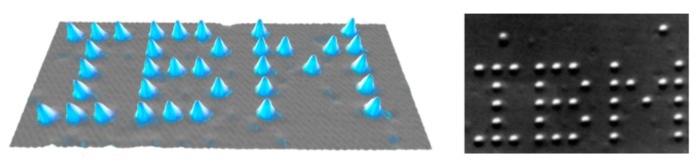
35 Xenon atoms positioned on a nickel (110) substrate using a STM to form IBM logo. Reproduced with permission from reference [23].

A few years later, in 1990, Don Eigler of IBM in Almaden and his colleagues used a STM to manipulate 35 individual xenon atoms on a nickel surface and formed the letters of IBM logo (Figure 7) [23]. The STM was invented to image surfaces at the atomic scale and has been used as a tool with which atoms and molecules can be manipulated to create structures. The tunneling current can be used to selectively break or induce chemical bonds. 

**Figure 8:**
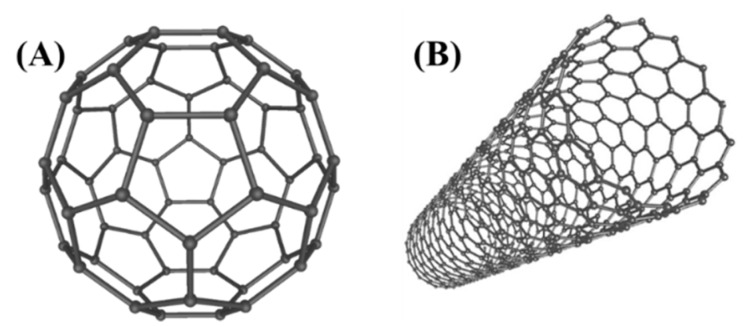
Schematic of a C60 buckyball (Fullerene) (**A**) and carbon nanotube (**B**).

In 1986, Binnig and Rohrer received the Nobel Prize in Physics “for their design of the STM”. This invention led to the development of the atomic force microscope (AFM) and scanning probe microscopes (SPM), which are the instruments of choice for nanotechnology researchers today [24,25]. At the same time, in 1985, Robert Curl, Harold Kroto, and Richard Smalley discovered that carbon can also exist in the form of very stable spheres, the fullerenes or buckyballs [26]. The carbon balls with chemical formula C60 or C70 are formed when graphite is evaporated in an inert atmosphere. A new carbon chemistry has been now developed, and it is possible to enclose metal atoms and create new organic compounds. A few years later, in 1991, Iijima et al. observed of hollow graphitic tubes or carbon nanotubes by Transmission Electron Microscopy (TEM) which form another member of the fullerene family (Figure 8) [27]. The strength and flexibility of carbon nanotubes make them potentially useful in many nanotechnological applications. Currently, Carbon nanotubes are used as composite fibers in polymers and beton to improve the mechanical, thermal and electrical properties of the bulk product. They also have potential applications as field emitters, energy storage materials, catalysis, and molecular electronic components.

In 2004, a new class of carbon nanomaterials called carbon dots (C-dots) with size below 10 nm was discovered accidentally by Xu et al. during the purification of single-walled carbon nanotubes [28]. C-dots with interesting properties have gradually become a rising star as a new nanocarbon member due to their benign, abundant and inexpensive nature [29]. Possessing such superior properties as low toxicity and good biocompatibility renders C-dots favorable materials for applications in bioimaging, biosensor and drug delivery [30,31,32,33,34,35]. Based on their excellent optical and electronic properties, C-dots can also offer exciting opportunities for catalysis, energy conversion, photovoltaic devices and nanoprobes for sensitive ion detection [36,37,38,39]. After the discovery of “graphene” in 2004, carbon-based materials became the backbone of almost every field of science and engineering.

In the meantime, nanoscience progressed in other fields of science like in computer science, bio and engineering. Nanoscience and technology progressed in computer science to decrease the size of a normal computer from a room size to highly efficient moveable laptops. Electrical engineers progressed to design the complex electrical circuits down to nanoscale level. Also, many advances are noticed in smart phone technology and other modern electronic devices for daily uses.

At the beginning of 21st century, there was an increased interest in the nanoscience and nanotechnology fields. In the United States, Feynman’s concept of manipulation of matter at the atomic level played an important role in shaping national science priorities. During a speech at Caltech on 21 January 2000, President Bill Clinton advocated for the funding of research in the field of nanotechnology. Three years later, President George W. Bush signed into law the 21st century Nanotechnology Research and Development Act. The legislation made nanotechnology research a national priority and created the National Technology Initiative (NNI).

Recently, a number of studies highlighted the huge potential that nanotechnologies play in biomedicine for the diagnosis and therapy of many human diseases [40]. In this regard, bio-nanotechnology is considered by many experts as one of the most intriguing field of application of nanoscience. During recent decades, the applications of nanotechnology in many biology related areas such as diagnosis, drug delivery, and molecular imaging are being intensively researched and offered excellent results. Remarkably, a plethora of medical-related products containing nanomaterials are currently on the market in the USA. Examples of “nanopharmaceuticals” include nanomaterials for drug delivery and regenerative medicine, as well as nanoparticles with antibacterial activities or functional nanostructures used for biomarker detection like nanobiochips, nanoelectrodes, or nanobiosensors [41].

One of the most important applications of nanotechnology to molecular biology has been related to nucleic acids. In 2006, Paul Rothemund developed the “scaffolded DNA origami”, by enhancing the complexity and size of self-assembled DNA nanostructures in a “one-pot” reaction [42]. The conceptual foundation for DNA nanotechnology was first laid out by Nadrian Seeman in 1982: “It is possible to generate sequences of oligomeric nucleic acids, which will preferentially associate to form migrationally immobile junctions, rather than linear duplexes, as they usually do” [43]. DNA nanotechnology has already become an interdisciplinary research area, with researchers from physics, chemistry, materials science, computer science, and medicine coming together to find solutions for future challenges in nanotechnology [44,45,46,47]. Notably, years of extensive studied made possible to use DNA and other biopolymers directly in array technologies for sensing and diagnostic applications.

Remarkable progresses have been made also in the field of nano-oncology by improving the efficacy of traditional chemotherapy drugs for a plethora of aggressive human cancers [48,49]. These advances have been achieved by targeting the tumour site with several functional molecules including nanoparticles, antibodies and cytotoxic agents. In this context, many studies showed that nanomaterials can be employed itself or to deliver therapeutic molecules to modulate essential biological processes, like autophagy, metabolism or oxidative stress, exerting anticancer activity [50].

Hence, nano-oncology is a very attractive application of nanoscience and allows for the improvement of tumour response rates in addition to a significant reduction of the systemic toxicity associated with current chemotherapy treatments.

Nanotechnology has been used to improve the environment and to produce more efficient and cost-effective energy, such as generating less pollution during the manufacture of materials, producing solar cells that generate electricity at a competitive cost, cleaning up organic chemicals polluting groundwater, and cleaning volatile organic compounds (VOCs) from air.

However, the application of computational approaches to nanomedicine is yet underdeveloped and is an exigent area of research. The need for computational applications at the nano scale has given rise to the field of nanoinformatics.

Powerful machine-learning algorithms and predictive analytics can considerably facilitate the design of more efficient nanocarriers. Such algorithms provide predictive knowledge on future data, have been mainly applied for predicting cellular uptake, activity, and cytotoxicity of nanoparticles.

Data mining, network analysis, quantitative structure-property relationship (QSPR), quantitative structure–activity relationship (QSAR), and ADMET (absorption, distribution, metabolism, excretion, and toxicity) predictors are some of the other prominent property evaluations being carried out in nanoinformatics.

Nanoinformatics has provided a major supplementary platform for nanoparticle design and analysis to overcome such in vitro barriers. Nanoinformatics exclusively deals with the assembling, sharing, envisaging, modeling, and evaluation of significant nanoscale level data and information. Nanoinformatics also facilitates chemotherapy by improving the nano-modeling of the tumor cells and aids detection of the drug-resistant tumors easily. Hyperthermia-based targeted drug delivery and gene therapy approaches are the latest nanoinformatics techniques proven to treat cancer with least side effects [51].

## 5. Conclusions

**Figure 9:**
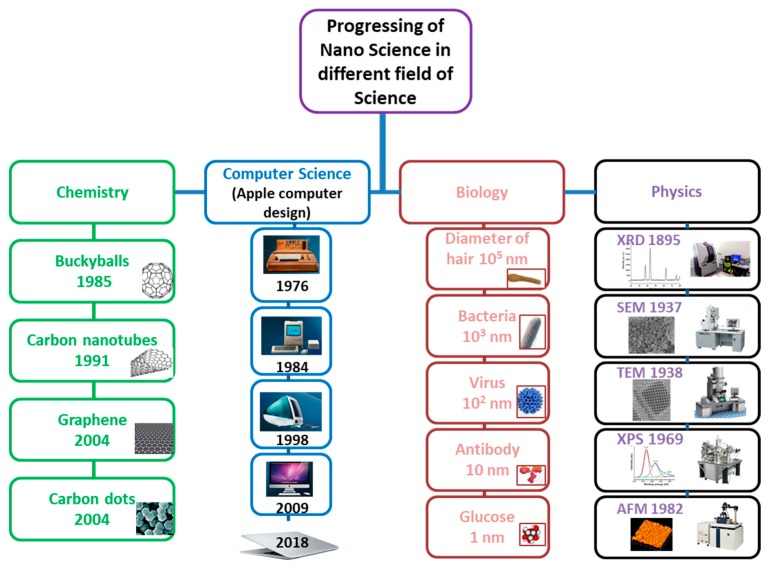
Progress in nanoscience and nanotechnology in different fields of science.

The progress of nanoscience and nanotechnology in different fields of science has expanded in different directions, to observe things from micro to nano, to even smaller scale sizes by different microscopes in physics, from micro size bulk matter to small size carbon dots in chemistry, from room size computers to mobile slim size laptops in computer science, and to observe deeply the behavior of the cell′s nucleus to study single complicated biomolecules at the nano level in biological science. All these progressions in different fields of science have been generally overviewed and summarized in Figure 9.

In only a few decades, nanotechnology and nanoscience have become of fundamental importance to industrial applications and medical devices, such as diagnostic biosensors, drug delivery systems, and imaging probes. For example, in the food industry, nanomaterials have been exploited to increase drastically the production, packaging, shelf life, and bioavailability of nutrients. In contrast, zinc oxide nanostructures display antimicrobial activity against food-borne bacteria, and a plethora of different nanomaterials are nowadays used for diagnostic purposes as food sensors to detect food quality and safety [52].

Nanomaterials are being used to build a new generation of solar cells, hydrogen fuel cells, and novel hydrogen storage systems capable of delivering clean energy to countries still reliant on traditional, non-renewable contaminating fuels.

However, the most significant advances in nanotechnology fall in the broad field of biomedicine and especially in cancer therapeutics because of their great potential to offer innovative solutions to overcome the limitations deriving by traditional chemotherapy and radiotherapy approaches.

Recent advances made in the fields of physic, chemistry and material sciences have provided a number of nanomaterials with unique properties, which are expected to improve the treatment of many tumors otherwise resistant to current therapies. This will be possible by merit of their intrinsic cytotoxic activity and/or because of their capability to act as nanocarriers to deliver therapeutic molecules, such as drugs, proteins, nucleic acids or immune agents. These innovative biomedical applications are currently exploited in a variety of clinical trials and, in the near future, may support major development in the therapy of cancer.

In 2018, the budget for NNI was 1.2 billion dollars ($) to support nanoscience, engineering and technology. Still, scientists are working for new breakthroughs in nanoscience and nanotechnology in order to make human life easier and more comfortable.

In this context, Table 1 presents the historical development of nanoscience and nanotechnology.

## Figures and Tables

**Table 1:** Evolution Timeline of Nanoscience and Nanotechnology.

Year	Event	References
4th Century	Lycurgus Cup (Colored glass).	[12]
500–1450	Cathedrals (Stained glasses windows).	[53]
1450–1600	Deruta Pottery (Iridescent/metallic clusters).	[53]
1857	Michael Faraday (Synthesis of colloidal ruby gold nanoparticles).	[18]
1908	Gustav Mie (Light scattering nanoparticles).	[54]
1928	Edward Synge (Near-field optical microscope).	[55]
1931	Max Knoll and Ernst Ruska (invention of transmission electron microscope (TEM)).	[56,57]
1936	Erwin Müller (Invention of field electron microscope).	[58]
1947	William Shockley, Walter Brattain and John Bardeen (Discovery of the semiconductor transistor).	[59]
1951	Erwin Müller (Invention of field-ion microscope, first to see atoms on the surface).	[60,61]
1953	James Watson and Francis Crick (Discovery of DNA).	[62]
1956	Arthur Von Hippel (Molecular Engineering).	[63]
1958	Leo Esaki (Electron tunneling).	[64]
1959	Richard Feynman (There’s Plenty of Room at the Bottom).	[5]
1960	Charles Plank and Edward Rosinski (Zeolites and catalysis).	[65]
1963	Stephen Papell (Invention of Ferrofluids).	[66]
1965	Gordon E. Moore (Moore’s Law).	[67]
1970	Eiji Osawa (Predicted the existence of C60 in the form of icosahedron).	[68]
1974	Norio Taniguchi (First use of the term “Nanotechnology”).	[6]
1974	Mark A. Ratner and Arieh Aviram (Molecular electronics).	[69]
1977	Richard P. Van Duyne (Discovery of Surface Enhanced Raman Spectroscopy (SERS)).	[70]
1980	Jacop Sagiv (Discovery of Self-Assembly Monolayers (SAMs)).	[71]
1981	Gerd Binnig and Heinrich Rohrer (Invention of Scanning Tunneling Microscope (STM)).	[72]
1981	Alexey Ekimov (Discovery of nanocrystalline Quantum Dots in a glass matrix).	[73]
1981	Eric Drexler (Molecular Engineering).	[74]
1982	Nadrian Seeman (Development of the concept of DNA Nanotechnology).	[43,75]
1983	Louis Brus (Discovery of colloidal Quantum Dots).	[76,77]
1985	Richard Smalley, Robert Curl and Harold Kroto (Discovery of Buckminsterfullerenes C60).	[26]
1986	Gerd Binnig, Christoph Gerber and Calvin F. Quate (Invention of Atomic Force Microscope (AFM).	[24]
1987	Dimitri Averin and Konstantin Likharev (Single-Electron Tunneling (SET) transistor).	[78]
1990	Donald Eigler and Erhard Schweizer (Arranged of individual Xenon atoms to form the letters IBM).	[23]
1991	Sumio Iijima (Discovery of Multi-wall Carbon nanotubes).	[27]
1992	Charles T. Kresge (Discovery of mesoporous silica MCM-41).	[79,80]
1993	Sumio Iijima and Donald Bethune (Discovery of Single-wall Carbon nanotubes).	[81,82]
1996	Chad Mirkin and Robert Letsinger (SAM of DNA+gold colloids).	[83]
1997	Zyvex (First nanotechnology company founded).	[84]
1998	Cees Dekker (Creation of a Transistor using carbon nanotubes).	[85]
1999	Chad Mirkin (Development of Dip-pen Nanolithography (DPN)).	[86]
2000	Mark Hersam and Joseph Lyding (Feedback-Controlled Lithography (FCL).	[87]
2000	President Bill Clinton announces US National Nanotechnology Initiative (NNI).	[88]
2001	Carlo Montemagno (Molecular nanomachines: molecular motor (rotor) with nanoscale silicon devices).	[89]
2002	Cees Dekker (Carbon nanotubes functionalized with DNA).	[90]
2003	President George W. Bush signed into law the 21st Century Nanotechnology Research and Development Act.	[91]
2003	Naomi Halas (Development of gold nanoshells).	[92,93]
2004	Andre Geim and Konstantin Novoselov (Discovery of graphene).	[94]
2004	Xu et al. (Discovery of Fluorescent Carbon dots).	[28]
2005	James Tour (Nanocar with turning buckyball wheels).	[95,96]
2006	Paul Rothemund (DNA origami).	[42]
2007	J. Fraser Stoddart (artificial molecular machines: pH-triggered muscle-like).	[97]
2008	Osamu Shimomura, Martin Chalfie and Roger Y. Tsien (Nobel Prize in Chemistry for the discovery and development of the green fluorescent protein, GFP).	[98]
2009	Nadrian Seeman (DNA structures fold into 3D rhombohedral crystals).	[99]
2010	IBM (Development of an ultra-fast lithography to create 3D nanoscale textured surface).	[100]
2011	Leonhard Grill (scanning tunneling microscope (STM) describes the electronic and mechanical properties of individual molecules and the polymer chains).	[101]
2016	Jean-Pierre Sauvage, Sir J. Fraser Stoddart and Bernard L. Feringa (Nobel Prize in Chemistry for the design and synthesis of molecular machines).	[102]
2017	Nobel Prize in Physics 2017: Gravitational waves.	[103]
2018	World’s smallest tic-tac-toe game board made with DNA.	[104]
2018	Shrinking objects to the nanoscale.	[105]
